# The Silkworm (*Bombyx mori*) microRNAs and Their Expressions in Multiple Developmental Stages

**DOI:** 10.1371/journal.pone.0002997

**Published:** 2008-08-20

**Authors:** Xiaomin Yu, Qing Zhou, Sung-Chou Li, Qibin Luo, Yimei Cai, Wen-chang Lin, Huan Chen, Yue Yang, Songnian Hu, Jun Yu

**Affiliations:** 1 Key Laboratory of Genome Sciences and Information, Beijing Institute of Genomics, Chinese Academy of Sciences, Beijing, China; 2 Graduate University of Chinese Academy of Sciences, Beijing, China; 3 James D. Watson Institute of Genome Sciences, Zhejiang University, Hangzhou, China; 4 Institute of BioMedical Informatics, National Yang-Ming University, Taipei, Taiwan; 5 Bioinformatics Program, Taiwan International Graduate Program, Academia Sinica, Taipei, Taiwan; 6 Institute of Biomedical Sciences, Academia Sinica, Taipei, Taiwan; Deutsches Krebsforschungszentrum, Germany

## Abstract

**Background:**

MicroRNAs (miRNAs) play crucial roles in various physiological processes through post-transcriptional regulation of gene expressions and are involved in development, metabolism, and many other important molecular mechanisms and cellular processes. The *Bombyx mori* genome sequence provides opportunities for a thorough survey for miRNAs as well as comparative analyses with other sequenced insect species.

**Methodology/Principal Findings:**

We identified 114 non-redundant conserved miRNAs and 148 novel putative miRNAs from the *B. mori* genome with an elaborate computational protocol. We also sequenced 6,720 clones from 14 developmental stage-specific small RNA libraries in which we identified 35 unique miRNAs containing 21 conserved miRNAs (including 17 predicted miRNAs) and 14 novel miRNAs (including 11 predicted novel miRNAs). Among the 114 conserved miRNAs, we found six pairs of clusters evolutionarily conserved cross insect lineages. Our observations on length heterogeneity at 5′ and/or 3′ ends of nine miRNAs between cloned and predicted sequences, and three mature forms deriving from the same arm of putative pre-miRNAs suggest a mechanism by which miRNAs gain new functions. Analyzing development-related miRNAs expression at 14 developmental stages based on clone-sampling and stem-loop RT PCR, we discovered an unusual abundance of 33 sequences representing 12 different miRNAs and sharply fluctuated expression of miRNAs at larva-molting stage. The potential functions of several stage-biased miRNAs were also analyzed in combination with predicted target genes and silkworm's phenotypic traits; our results indicated that miRNAs may play key regulatory roles in specific developmental stages in the silkworm, such as ecdysis.

**Conclusions/Significance:**

Taking a combined approach, we identified 118 conserved miRNAs and 151 novel miRNA candidates from the *B. mori* genome sequence. Our expression analyses by sampling miRNAs and real-time PCR over multiple developmental stages allowed us to pinpoint molting stages as hotspots of miRNA expression both in sorts and quantities. Based on the analysis of target genes, we hypothesized that miRNAs regulate development through a particular emphasis on complex stages rather than general regulatory mechanisms.

## Introduction

As key regulators for gene expression at post-transcriptional levels, microRNAs (miRNAs) are a class of endogenous non-coding RNAs transcribed by RNA polymerase II with a size range of ∼22 nt (nucleotides); they are processed from larger hairpin structures—known as pri- and pre-miRNAs—by two specialized proteins, namely Drosha and Dicer (or Dicer-like proteins, DCLs) [1], [2]. Mature miRNAs are incorporated into the RNA-induced silencing complex (RISC) with the Piwi/Argonaute (AGO) protein family to exert their functions [3], whereas their opposite strands, known as miRNAs*, are scrapped [4], [5]. Previous discoveries suggested that the strand whose 5′ end is less stably paired as miRNA:miRNA* duplex is preferentially packaged into RISC [6], [7]. Similar to *lin-4* and *let-7* of *Caenorhabditis elegans*, the majority of miRNA genes are transcribed as independent transcriptional units [4], [8], [9] albeit a few of them were found in introns of pre-mRNAs and co-expressed with their host genes [5], [10], [11], [12], [13]. A large fraction of miRNAs are conserved among closely-related species, and many even have homologs in distant species. For instance, more than a third of miRNAs found in *C. elegans* have homologs in humans [5], suggesting that they have important functions that are evolutionarily-conserved [14]. miRNA genes are sometimes observed as clusters frequently transcribed as single polycistronic transcript [2], [10], implying that they may share common functional relationships. Since *lin-4* and *let-7* were found to regulate development in *C. elegans* [15], [16], ample reports have suggested that miRNAs play significant regulatory roles under physiological (such as development) and pathological (such as cancers) conditions in plants, flies, fishes, and mammals [14], [17], [18], [19], [20], [21]. Although animal miRNAs tend to show imperfect base-pairing in 3′ untranslated regions (3′ UTRs) of their target transcripts, a 7-nt seed sequence starting from the second nucleotide at the 5′ end of miRNAs pairs specifically with their target mRNA is reported to lead to decreased transcription and/or translational repression [11], [22], [23], [24], [25]. Viruses also use their miRNAs to repress host genes that include transcriptional regulators, components of signal transduction pathways, B cell-specific chemokines, and cytokines [26], [27], [28].

Currently, 5071 miRNAs have been annotated in miRBase (release 10.0) (http://microrna.sanger.ac.uk/sequences/) identified by either experimental or computational approaches [29]. For instance, direct sequencing and Northern blotting validations for endogenous small RNAs have been successful in identifying the majority of known miRNAs [26], [30], [31], [32], [33], [34], [35], [36], [37], [38]. One limitation of these approaches is its poor detectability for both low abundant miRNAs due to variable expression levels and their specificities of precise temporal and spatial expressions during developmental stages. For instance, in *Drosophila melanogaster*, *mir-3*, *mir-4*, *mir-5,* and *mir-6* are down-regulated in the transition from embryo to larva, whereas *mir-100*, *mir-125,* and *let-7* are up-regulated from larva to pupa [39]. Another example is the fact that miRNAs are often expressed in a strict tissue-specific manner during development; for instance, miR-124a is expressed only in brain and spinal cord of vertebrates and ventral nerve cord of insects [40], [41], [42]. Other examples are miR-1, miR-133, and miR-206; all are strictly expressed in muscles and heart [43], [44], [45], [46].

Taking the advantage of computational algorithms that have been developed based on structural characteristics of miRNA precursors and phylogenetic conservation for the prediction of miRNAs [5], [11], [37], [47], [48], [49], [50], [51] as well as the recently-sequenced domesticated silkworm (*B. mori*) genome [52], [53], we studied miRNAs in this economically important species by using a combined strategy—computational prediction and experimental identification. For the computational approach, we added custom-designed filters based on known characteristics of insect miRNAs in addition to software tools, such as *Srnaloop* [48]. For the experimental approach, we initially made 14 small-RNA-specific cDNA libraries for the selected developmental stages throughout the life cycle of silkworms from pre-diapaused egg (embryo) to larva and moth, sequenced adequate amounts of clones from the libraries, and confirmed in part our *in silico* discoveries and predictions. We employed the stem-loop RT PCR to confirm the cloned novel miRNAs and analyzed the expression pattern of selected miRNAs in 14 developmental stages. In the context of predicted target genes and life history traits of silkworms; we also discussed potential functions of several stage-biased miRNAs.

## Results

### Computational identification of conserved miRNAs and novel pre-miRNA candidates

We first surveyed the *B. mori* genome assembly to predict candidate pre-miRNAs using *Srnaloop* program and custom-designed filters (see Materials and Methods for details), we then searched Rfam database [54], an insect ncRNA dataset, and a CDS dataset assembled from the silkworm genome for removing those overlapping with these specialized sequences, yielding 7,241,352 candidate pre-miRNAs. Since most of the mature miRNAs are evolutionarily-conserved among diverged species [5], we first identified hairpin sequences from *B. mori*, which are homologous to known metazoan miRNAs by searching miRBase 10.0 [29], and identified 114 distinct conserved miRNAs. Of them, there were 21 previously-known miRNAs from *B. mori* [52] (Table S1). 78 were classifiable into known families based on their precursor sequences, and 45 have homologs among known insect miRNAs. We also discovered six pairs that are organized as clusters; bmo-miR-1/bmo-miR-133, bmo-let-7/bmo-miR-100, bmo-miR-12/bmo-miR-283, and bmo-miR-275/bmo-miR-305 are separated by less than 20 kb apart and in the same orientation; bmo-miR-9b overlaps with bmo-miR-79 on the opposite strand; and bmo-miR-2 is adjacent to bmo-miR-13 but on the reverse strand in a tail-to-tail orientation about some twenty basepairs away. Most strikingly, there are six members of bmo-miR-466 family, and three have multiple copies. For instance, bmo-miR-466e has 16 copies in the current silkworm genome assembly.

For predicting novel pre-miRNA candidates, we used filters based on sequences and structure features to limit false positives, including GC content as well as minimum free energy of entire hairpins, and core hairpin structures. To evaluate the performance of our pipeline, we carried out a sensitivity test. Starting from 279 insect pre-miRNAs found in five known insect genomes, we obtained 279 and 252 pre-miRNAs after folding and filtering procedures, yielding sensitivities of 100% and 90.3%, respectively (Table 1).

**Table 1:** Sensitivity test on 279 published insect pre-miRNAs.

Tested miRNAs	Number of miRNAs	Number of miRNAs after *Srnaloop*	Number of miRNAs after filters
*A. gambiae*	38	38	37
*A. mellifera*	54	54	43
*B. mori*	21	21	18
*D. melanogaster*	93	93	84
*D. pseudoobscure*	73	73	70
Total (Sensitivity)	-	279 (100%)	252 (90.3%)

We also selected candidates whose sequences and structure features are consistent with reference range values as listed in Table 2. In addition, we searched the sequences against genomic sequences of *D. melanogaster*, *Drosophila pseudoobscure*, *Apis mellifera, Anopheles gambiae* as well as the silkworm ESTs. Our procedure yielded 148 novel non-redundant pre-miRNAs candidates that are anchored to the genome sequence and tailored with relative abundances although in most cases only one arm of these pre-miRNAs is processed into mature miRNAs and conserved among closely related species. Our result indicated that conserved arms (3′ arm or 5′ arm) in closely related species are more likely to harbor authentic miRNAs. For instance, S1 has a conserved 3′ arm among *D. melanogaster*, *D. pseudoobscure*, *A. mellifera* and *A.gambiae*, better than its 5′ arm. We also found that 28 candidates (∼18.9%) matched silkworm ESTs; shows that they are transcribed, which is consistent with them being real miRNAs (Table S2).

**Table 2:** Distributions and optimal ranges of quantifiable features of pre-miRNAs.

	GC content	Core mfe^a^	Hairpin mfe^b^	Ch_ratio^c^
Distribution	16–70	−32.1– −8.1	−46.3– −12	0.39–0.99
Reference value	30–60	−29.7– −10	−40– −15	0.4–0.99

a Minimum free energy of the whole hairpin.

b Minimum free energy (mfe) of the core of hairpin structure

c Ratio of core mfe to hairpin mfe.

### Cloning and identification of silkworm miRNAs

We also took a direct-cloning approach to identify novel miRNAs. We first constructed 14 independent small RNA libraries (in an insert-size range of 15 to 40 nucleotides) across the life span (from fertilized eggs to pre-diapaused embryos and all the way to moth; see Materials and Methods) of silkworms. We acquired 6,720 clones (480 clones from each library) and 3,721 sequences are in a length range of 16 to 40 nucleotides; 69% of them have at least one match in the silkworm genome sequence annotated in SilkDB (http://silkworm.genomics.org.cn/) and the database posted by the silkworm genome research program (http://sgp.dna.affrc.go.jp/index.html). The remaining 31% did not match anything and were not analyzed further. Among the cloned small RNAs, we also identified rRNA (9%), tRNA (5%), sn/snoRNA (1%), and other non-coding RNAs (4%) as well as a small fraction (2%) that contains breakdown products of mRNAs. 2% of the sequences are believed to be putative miRNAs (Table 3), and the remaining 46% of short sequences failed to be classified based on the current silkworm genome sequence assembly.

**Table 3:** miRNAs expressed in *B. mori* a.

Name	Matched prediction candidates^c^	Sequence (5′ to 3′)	Total clones	PDS	DS	DBS	BKS	TAS	BS	NLS	FLS	MLS	LFLS	SS	PPS	PS	MS
bmo-miR-1	H2	UGGAAUGUAAAGAAGTGTGGA	1												1		
bmo-miR-7	H92	UGGAAGACUAGUGAUUUUGUUGU	1											1			
bmo-miR-8	H101	UAAUACUGUCAGGUAAAGAUGUC	3										1		2		
bmo-miR-9a	H110	UCUUUGGUUAUCUAGCUGUAUGA	1	1													
bmo-miR-10	H3	UACCCUGUAGAUCCGAAUUUGU	2							2							
bmo-miR-13	H11	UAUCACAGCCAUUUUUGACGAGUU	2										1			1	
bmo-miR-31	H39	GGCAAGAAGUCGGCAUAGCUGU	2									2					
bmo-miR-71	H95	UGAAAGACAUGGGUAGUGAGAUGU	1											1			
bmo-miR-71*	H95	UCUCACUACCUUGUCUUUCAUG	1												1		
bmo-miR-77	H99	UCAUCAGGCCAUAGUUGUCCA	1	1													
bmo-miR-92	H104	AAUUGCACCAAUCCCGGCCUGC	1	1													
bmo-miR-263a^b^	H22	AAUGGCACUGGAAGAAUUCACGGG	5						1			4					
		AAUGGCACUGGAAGAAUUCACGG	5									3		2			
		AAUGGCACUGGAAGAAUUCACG	11								1	7		3			
		AUGGCACUGGAAGAAUUCACG	1									1					
		AAUGGCACUGGAAGAAUUCA	4									4					
bmo-miR-263a*	H22	CUCUUAGUGGCAUCAC	1						1								
bmo-miR-263b	H23	CUUGGCACUGGGAGAAUUCA	1									1					
bmo-miR-275	H24	UCAGGUACCUGAAGUAUCGCG	1									1					
bmo-miR-278	–	UCGGUGGGAUCUUCGUCCGUUU	1									1					
bmo-miR-279	H27	UGACUAGAUCCACACUCAU	2			1											1
bmo-miR-282*^b^	H29	GACAUAGCCUGAUAGAGGUUACG	2									1	1				
		ACAUAGCCUGAUAGAGGUUACG	1							1							
bmo-miR-285	H31	UAGCACCAUUCGAAUUCAGUGC	4				1		1	1						1	
bmo-miR-306	–	UCAGGUACUAGGUGACUCUGA	3									2	1				
bmo-miR-317^b^	–	AGUGAACACAGCUGGUGGUAU	2									2					
		UGAACACAGCUGGUGGUA	1										1				
bmo-miR-768	–	GAGGAUGAAAUUAUCGAGCUAC	1												1		
bmo-miR-iab-4-3p	H113	CGGUAUACCUUCAGUAUACGUAAC	1											1			
bmo-miR-1920	–	GCGUGCGCGUAGCGAGUUC	1									1					
bmo-miR-1921	–	UGAGAUUCAGCCUUGCGCCAGGU	1												1		
bmo-miR-1922	–	GUUCGUCGUGGAUUUAAGA	1									1					
bmo-miR-1923	S105	UAAUCGCGUACCGUUGCAUAGCCGUGGC	1									1					
bmo-miR-2008^b^	S147	CGGCGAGAGGGACGCUCCUUAGAGUCG	1					1									
		AGGGACGCUCCUUAGAGUCGGGUU	1					1									
		GCGAGAGGGACGCUCCUUAGA	1			1											
bmo-miR-2009	S127	GACCCGAAAGAUGGUGA	1			1											
bmo-miR-2009*	S127	GAUGGAGGAUCGUAGCA	1						1								
bmo-miR-2010	S140	CACCACGGAAACACAAUAAUUG	1												1		
bmo-miR-1926	S104	AGGAAUUCUAAAGCAAAAAGG	1														1
bmo-miR-2007	S122	UAAAAACGUGCGUUGGCCG	1											1			
bmo-miR-1924	S45	UGAUGUCCGCGGAGGUGUAGUG	1									1					
bmo-miR-1925	S20	UUUUCAACAUGGUAUGGAC	1		1												
Total clones			77	3	1	3	1	2	4	4	1	33	5	9	7	2	2

a Pre-diapaused egg(PDS); Diapaused egg(DS); Diapause-broken egg(DBS); Blastokinesis stage egg(BKS); Trachea appearing stage egg(TAS); Bluish egg(BS); Newly-hatched larva(NLS); Fourth-instar larva(FLS); Molting larva(MLS); Late fifth-instar larva(LFLS); Spinning larva(SS); Pre-pupa (PPS); Pupa (PS); Moth (MS).

b The sequences have length heterogeneity found on the 5′ and/or 3′ end, and different mature forms from the same stem of precursor are also listed.

c H, ID of miRNAs predicted based on homolog conservation comparison with known Metazoa miRNAs; S, ID of putative miRNAs predicted based on Sequence & Structural features filters.

**Figure 1:**
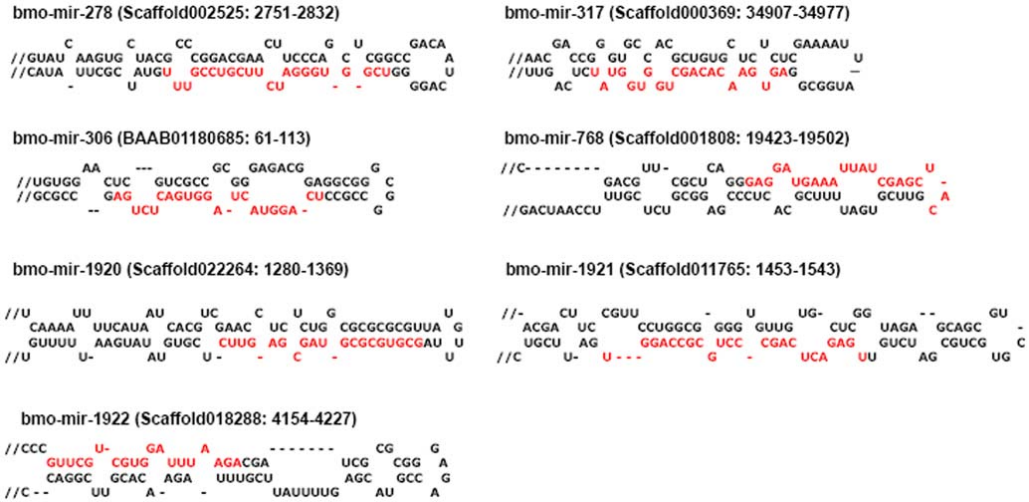
Predicted stem-loop structures of precursors and mature forms (highlight in red) of newly-identified miRNAs based on our direct cloning approach from the silkworm. Hairpins longer than 120 nt were truncated; these hairpins are indicated by a double slash preceding the stem. Scaffold numbers and genomic positions are indicated in parentheses.

**Figure 2:**
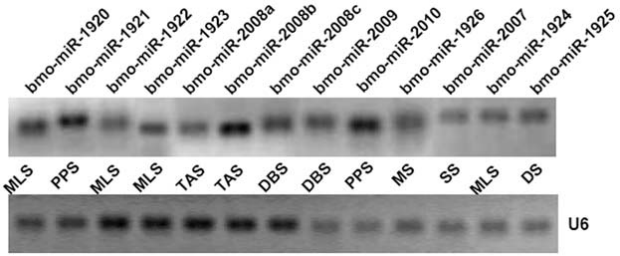
Expression confirmation of novel miRNAs identified by cloning from specific development stages of silkworm using stem-loop RT PCR. For bmo-miR-2008, we detected its three different mature segments, bmo-miR-2008a, bmo-miR-2008b and bmo-miR-2008c; for bmo-miR-2009, we detected only the mature segment from 5′ arm of its precursor. U6 snRNA serves as a positive control.

Our sequence analyses on the sequenced clones have yielded 55 miRNAs, representing 17 unique conserved miRNAs already discovered computationally. We also found several miRNA*s, such as bmo-miR-263a* in bluish egg (BS), bmo-miR-71* in spinning larva (SS) and pre-pupa (PPS), which providing solid evidence for Dicer-like processing [55], [56], [57], as it was reported that miRNA*s can also be functional [43], [58], [59]. Another group of 11 clones representing 11 novel putative miRNAs in our predicted data were confirmed by direct cloning (Table 3). In addition, four highly-conserved miRNAs (bmo-miR-278, bmo-miR-306, bmo-miR-317 and bmo-miR-768) and three less-conserved miRNAs (bmo-miR-1920, bmo-miR-1921 and bmo-miR-1922) were also cloned directly, which were not predicted based on our predicting criteria albeit their canonical precursors (Figure 1). To validate the novel miRNAs identified through direct cloning (bmo-miR-1920—bmo-miR-1926 and bmo-miR-2007—bmo-miR-2010), we deployed a stem-loop RT-PCR to assess the expression of these miRNAs (except bmo-miR-2009*). Expression of all 13 novel miRNAs at the different developmental stages was confirmed (Figure 2).

**Figure 3:**
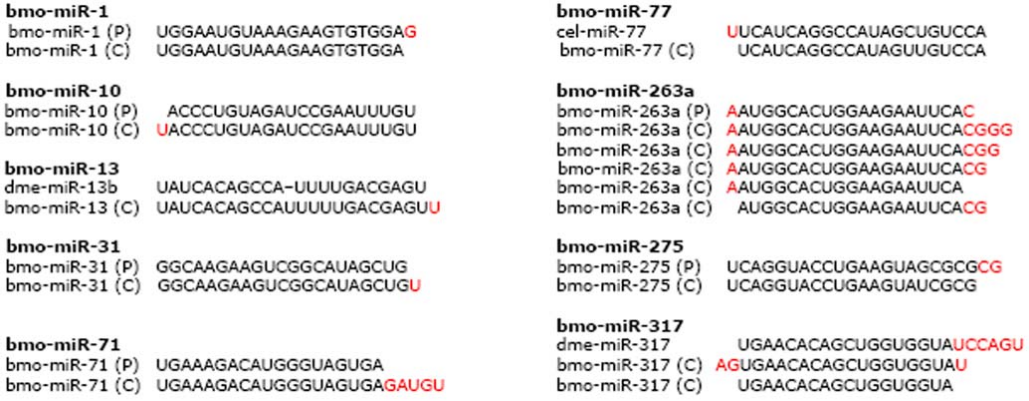
Length heterogeneity is found in the 5′ and/or 3′ end, the cloned sequences (C) of bmomiR-1, bmo-miR-10, bmo-miR-13, bmo-miR-31, bmo-miR-71, bmo-miR-77, bmo-miR-263a, bmo-miR-275 and bmo-miR-317 have nucleotides difference (highlighted in red) in 5′ and/or 3′ ends of the sequences as compared with their predicted sequences (P) or homologs among closely related species.

**Figure 4:**
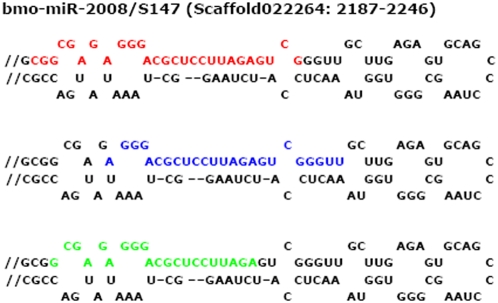
Three different segments of cloned bmo-miR-2008 (highlight in red, blue and green respectively) derived from the 5′ arm of its predicted pre-miRNA S147. Scaffold number and genomic positions are indicated in parentheses.

Direct cloning allowed us to identify several mature miRNAs sequences, such as bmo-miR-263a, bmo-miR-282*, and bmo-miR-317, which vary in their 5′ and/or 3′ ends on the same stem of their precursors (Table 3). It is difficult to recognize accurate sequences of mature miRNAs solely by computational prediction and Northern blotting. Direct sequencing provides solid evidence, to the level of single nucleotide. For instance nine miRNAs in our dataset (bmo-miR-1, bmo-miR-10, bmo-miR-13, bmo-miR-31, bmo-miR-71, bmo-miR-77, bmo-miR-263a, bmo-miR-275, and bmo-miR-317) have nucleotide differences in 5′ or/and 3′ end of their sequences when compared the mature forms to their predicted sequences or homologs in closely related species (Figure 3). Moreover, we found that bmo-miR-2008 from the 5′ stem of putative pre-miRNA S147 yields three different segments as mature miRNAs (Figure 4).

Based on the numbers of sequenced clones per library, assuming unbiased cloning process, miRNA expression profiles in *B. mori* appeared stage-biased. We detected expressions of miRNAs in multiple developmental stages, and the frequency of any single miRNA appearing in sequenced libraries varied from one to seven (Table 3). For instance, aside from a few commonly expressed miRNAs, such as bmo-miR-8, bmo-miR-13, bmo-miR-263a, bmo-miR-275, bmo-miR-279, bmo-282*, bmo-miR-285 and bmo-miR-306, most of them were found development-related (Table 3) although some may be due to inadequate sampling that often results less reliable statistics. Furthermore, we noticed that the highest number of miRNAs, both in sorts and quantities, were detected in the library made from RNA isolated at molting larva stage (MLS)—33 out of 77 sequences, representing 12 different miRNAs.

### Analyzing 15 mature miRNAs expression patterns and potential targets

**Figure 5:**
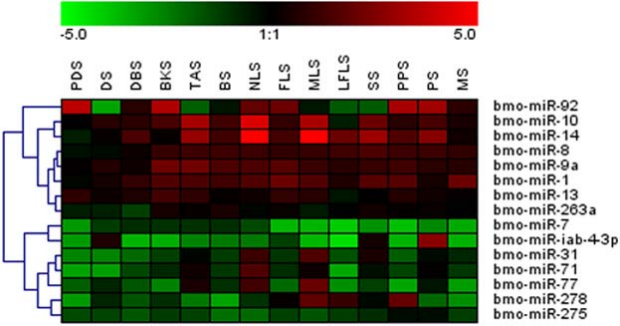
Expression profiles of 15 mature miRNAs in 14 developmental stages. Data from each miRNA were showed as dendrograms indicating expression correlation among genes. Samples and miRNAs are displayed in rows and columns, respectively. The relative expression values ranged from +5 log_10_ to −5 log_10_.

We analyzed the expression of 15 mature miRNAs from 14 different stages with stem-loop RT-PCR (Figure 5). The expression of bmo-miR-92 varied across the 14 developmental stages, highly expressed at five stages—pre-diapaused egg (PDS) (*p* = 0.0014), diapause-broken egg (BKS) (*p* = 0.0011), pupa (PS) (*p* = 0.0017) and PPS with slightly less significance (*p* = 0.067)—but lower in other stages, and the lowest at diapaused egg (DS) stage (*p* = 0.035). Bmo-miR-10 and bmo-miR-14 showed a synchronized trend where a sharp increase at trachea appearing stage (TAS) (*p* = 0.0048 and 0.0031, respectively), NLS (*p* = 0.011 and 0.00007, respectively), and MLS (*p* = 0.012 and 0.0012, respectively). Bmo-miR-31, bmo-miR-71, and bmo-miR-77 displayed a very similar expression pattern, higher expression at NLS (*p* = 0.027, 0.035 and 0.0064, respectively); bmo-miR-31 and bmo-miR-77 are also expressed at a higher level at MLS than at other stages (*p* = 0.019 and 0.0082, respectively). Bmo-miR-8, bmo-miR-9a, and bmo-miR-263a, similar to bmo-miR-1, showed a slight elevation after diapause-broken stage (DBS) and kept a relatively constant level thereafter. In the case of bmo-miR-278 and bmo-miR-iab-4-3p, our results showed that they displayed distinct increases at MLS (*p* = 0.022), late fifth-instar larva (LFLS) (*p* = 0.048), PPS (*p* = 0.042), and DS (*p* = 0.0083), SS (*p* = 0.012), PS (*p* = 0.022), respectively. The expression of bmo-miR-7 and bmo-miR-275 maintained at a relatively stable level in 14 development stages except bmo-miR-275 alone displayed a weak increase at both PPS and PS. Bmo-miR-13 showed fluctuating trend with its lowest level at LFLS (*p* = 0.042). In general, most of the miRNAs exhibited the highest expression at NLS and MLS (Spearman's *ρ* = 0.65, *p* = 0.01) but the lowest expression at DS; these results are in accord with the cloning results for the MLS, where abundant miRNA expression was observed.

We used the PITA program to identify the target genes of the 15 mature miRNAs with a default criterion and ΔΔG≤−5 kcal/mol. From the long list of the target genes, we selected potential targets with high probability after taking both expression patterns of miRNAs and silkworm life traits into account, the targets predicted or confirmed in *Drosophila* previously were also listed (Table 4). These target genes involved those of hormone-regulated pathways (such as myosuppressin receptor/BMSR, juvenile hormone esterase/Jhe, juvenile hormone acid methyltransferase/Jhamt, allatostatin receptor, bombyxin and RXR type hormone receptor/BmCF1), cell cycle control (such as inhibitor of apoptosis protein/*Iap*, annexin/EN16b, SCF apoptosis response protein/SCF, and serine/threonine protein phosphatase 6), signal transduction (such as notch homolog, presenilin enhancer, and achaete-scute-like protein/ASE), and binding (such as DNA supercoiling factor and RNA unwinding factor/*vas*).

**Table 4:** High-probability targets of 15 silkworm miRNAs.

No	miRNAs	Predicted targets (Known)*	Predicted targets (Novel)
1	bmo-miR-1	*Hr46*, HDAC4, Delta1	BMSR, Cjhbp, Jhe, eclosion hormone, dopa decarboxylase
2	bmo-miR-7	*Aop*, *HLHm3*, *Tom*,YAN, *hairy*	Ptsp, ecdysteroid-regulated 16 kDa protein precursor, *vas*, dopa decarboxylase, Jhamt, SCF apoptosis response protein, presenilin enhancer, BMSR, Ago2, Bras1
3	bmo-miR-8	*Ptp99A*, *atrophin*,	Lpr4, Cdc2, BmCF1, Bras1, *stathmin*, Pbanr, Jhamt, trehalase
4	bmo-miR-9a	*Br*, *Hr46*, *SOPs*	ASE, Ago2, chiB4, Jhe, thymosin isoform 1, BRFa, Notch homolog, *stathmin*,
5	bmo-miR-10	*Scr*, HOXA3	E75, BmCF1, ecdysone receptor, Jhe, *Eck*, EN16b
6	bmo-miR-13	*Rpr*, *hb*,	abnormal wing disc-like protein, chiB4, *Lysp*, *Scr*, MOF protein, Adamts-like protein, heptahelical receptor, Cjhbp, BMSR, *Iap*, notch-like protein
7	bmo-miR-14	*Br*, *NetA*, *EcR*, *Eip93F*, *Drice*	E75, BmBRC, BmCF1, BmCyc b, Jhe, Sgf-1
8	bmo-miR-31	*NetB*, *grim*, *reaper*, *sickle*	presenilin enhancer, Adamts-like protein, Ago2, Bras2, allatostatin preprohormone, eclosion hormone, Cjhbp
9	bmo-miR-71	-	*pbp2*, Ago2, ecdysone 20-hydroxylase, ecdysteroid-phosphate phosphatase, Pbanr, BRFa
10	bmo-miR-77	-	SCF apoptosis response protein, Jhe, *Iap*, eclosion hormone, *septin*, Jhamt, 20-hydroxy-ecdysone receptor, bombyxin
11	bmo-miR-92	*Hr96*	Eck, myosin light polypeptide, BmCyc b, tropomyosin isoform 2, *Boceropsin*, DNA supercoiling factor, EN16b
12	bmo-miR-263a	*Abd-A*, *Clk*, *dbt*,*tws*, *slo*	presenilin enhancer, calreticulin, ecdysteroid-regulated 16 kDa protein precursor, *Scr*, allatostatin receptor, dopamine transporter, caspase-1, allatostatin receptor
13	bmo-miR-275	*Scr*, *Drl-2*, *NetA*,	Bras2, Wcp4, presenilin enhancer, Pbanr, Rack1, BIR, cell death-regulatory protein
14	bmo-miR-278	*expanded*	BIR, Jhamt, *Iap*, ecdysone receptor, allatostatin preprohormone, allatostatin receptor, Rieske-domain protein Neverland, BmCF1, Eck, thymosin isoform 2, chitinase-like protein
15	bmo-miR-iab-4-3p	*-*	BmDHR-2, cell death-regulatory protein, BMSR, serine/threonine protein phosphatase 6, PKG-II, Sgf-1, allatostatin preprohormone, dopa decarboxylase, Adamts-like protein, IPPI_Bm

* Those target genes have been predicted or confirmed in *Drosophila*.

## Discussion

### Performance of our prediction procedures

Our prediction protocol took the advantage of previous studies [48], [60] as well as custom-designed conditional filters. In particular, we used shared features of insect mature miRNAs and pre-miRNAs, and the protocol gave rise to a sensitivity of 90.3% for known insect pre-miRNAs. However, several pre-miRNAs were not detected due to the stringency of the filters but discovered with our direct cloning experiments, including bmo-miR-278, bmo-miR-306, bmo-miR-317, bmo-miR-768, bmo-miR-1920, bmo-miR-1921, and bmo-miR-1922. Therefore, a combined approach is of essence to identify more miRNAs in any species even when genomic sequence is available and of high quality.

### miRNA clusters in silkworms

Six pairs of clustered miRNA genes were uncovered despite the fragmented nature of the draft sequence assembly. We noticed that the bmo-miR-1/bmo-miR-133 pair is highly conserved across diverse taxa including not only insects, such as honey bee and red flour beetle (data unpublished), but also vertebrates, such as frog, chicken, mouse, and human. In the silkworm genome assembly, this pair is situated on the antisene strand between the mind bomb homolog 1 (*MIB 1*) and CG30492-PC, but in other species, they are all exclusively harbored by the antisense strand of the same intron (intron12) of *MIB1*. *MIB1* as a ubiquitin ligase interacts with the intracellular domain of Delta and promoting its ubiquitylation and internalization for efficient activation of Notch pathway [61]. In *Drosophila*, miR-1 functions in Notch pathway through targeting the Notch ligand, Delta [62]. In frog embryos, miR-1 promotes muscle differentiation by targeting histone deacetylase4 (HDAC4) that acts as a transcriptional repressor in the Notch pathway [46]. These findings give us clues that conservative co-localization of the miR-1/miR-133 cluster and its antisense gene may exhibit their conserved functions in various species. Another pair of interesting clustered miRNAs in the silkworm genome contains bmo-miR-2 and bmo-miR-13, which are classified into the miR-2 family based on sequence similarity. Those two miRNAs also form a cluster in *D. melanogaster* and have been defined as proapoptotic K-box family miRNAs for regulating Notch target genes and K-box containing genes such as proapoptotic genes *grim*, *reaper*, and *sickle* [11], [23], [63]. The miR-2 family has been implicated in the control of apoptosis [23], [64]. Coincidently we detected bmo-miR-13 in two stages: late fifth-instar larva (LFLS) and pupa stages (PS), and its potential targets also include notch-like gene and inhibitor of apoptosis gene (*Iap*) (Table 4) suggesting that bmo-miR-2/13 have similar functions in silkworms and fruitflies.

### Imprecise and alternative cleavage of Dicer and origins of new functions for miRNAs

Some of the conserved miRNAs reported here have nucleotide difference in their 5′ and/or 3′ ends as compared with predicted sequences or homologs in closely-related species. Especially in bmo-miR-263a (Figure 3), changes of one nucleotide in 5′ or more in 3′ ends may not affect their regulatory roles because a mechanism for selecting target genes is based on nucleotide shuffling of a 7-nt seed sequence starting from the second nucleotide at the 5′ end of miRNAs. Such end polymorphism of miRNAs has also been observed by others using small RNA cloning approach [43], [65] and other methods, such as RNA-primed Array-based Klenow Extension (RAKE) [31]. The 5′ and/or 3′ heterogeneity might be mainly attribute to the less precise Drosha/Dicer processing, degradation at the 5′ and/or 3′ end and addition of untemplated nucleotides to the 3′ ends of miRNAs [56], [66]. In silkworms, such changes may occur in a similar ways as in other well-studied species for better performances in regulating their target genes. Although there has not been evidence to explain the functional implication of the sequence heterogeneity at 5′ and/or 3′ ends, our findings may support the idea that such nucleotide changes possibly affect the stability/subcellular localization of miRNAs and/or alter chemically dynamic parameters of miRNA-target interactions, thus induce miRNAs to select new target genes [25], [66]. The sufficient variations flank mature miRNAs could contribute to the evolutionary diversification of these key regulatory genes [67]. In our collection, for instance, the sequence of bmo-miR-2008 has three different mature forms deducible from the same stem of its precursor S147 (Figure 3); this phenomenon supports the possibility that imprecise and alternative cleavage during Dicer processing of mature miRNAs may allow miRNAs to acquire new functions. However, functional validation is needed to convince such active roles of Dicer contributing to the evolution of miRNAs.

### Stage-biased miRNAs and their potential functions

Our direct cloning approach served two basic purposes: discovering new miRNA candidates and obtaining rough frequencies in a per library manner. Our results offer indications of stage-biased expression of miRNAs in *B. mori*. In particular the most abundant miRNAs are found in NLS and MLS. Stage-biased miRNAs, such as bmo-miR-92 at PDS, bmo-miR-10 at NLS and MLS, bmo-miR-iab-4-3p at DS, SS and PS, may play crucial roles in regulating development-related genes. These stage-biased miRNAs provide insights into the mechanisms in regulating the developmental stages of *B. mori*.

As shown in Figure 5, bmo-miR-iab-4-3p exhibited a relatively high expression level at DS and SS, reaching its expression peak at PS (*p* = 0.022). We noticed that its predicted target genes—BMSR and cell death-regulatory protein—may be suppressed by bmo-miR-iab-4-3p at PS, resulting in normal level of ecdysone and inducing apoptosis of larval obligatory tissues as well as differentiation of imaginal discs at PS. Remarkably, bmo-miR-iab-4-3p displayed a relatively high level at DS whereas all other 14 miRNAs kept a low level expression. Previous research indicated silkworm cells are suspended in G2 at DS [68], [69], and Ser/Thr protein phosphatase, targeted by bmo-miR-iab-4-3p, controls the transition of G2 to M [70], suggesting that bmo-miR-iab-4-3p may play a pivotal role in keeping embryonic cells at G2 by suppressing the function of Ser/Thr protein phosphatase. In addition, dme-miR-92a, the ortholog of bmo-miR-92, was found expressed in the brain primordium and a subset of the ventral nerve cord in *Drosophila* [42], implying its possible role in regulating brain and nerve development. We also found *Boceropsin*, an important cerebral opsin of silkworms [71], is a potential target of bmo-miR-92. The relatively low expression of bmo-miR-92 at TAS and bluish egg stage (BS) may be essential for the function of *Boceropsin* and normal development of stemma.

The expression patterns of bmo-miR-10 and bmo-miR-14 are very similar, and they also shared several common targets, such as ecdysone receptor (*EcR*), orphan nuclear receptor (E75), BmCF1, and Jhe. Varghese and Cohen have reported that miR-14 plays a key role in modulating the positive autoregulatory loop by which ecdysone sensitizes its own signaling pathway [72]. Aside from *EcR*, bmo-miR-10 and bmo-miR-14 may together down-regulate the expression of Jhe, inducing juvenile hormone to accumulate slowly at late MLS for normal larva development after ecdysis.

Molting stage is composed of a series of successive processes including hypodermal cells activating, ecdysial fluid secreting, cuticular chitin and exoskeleton degrading, new epidermis formation, and old epidermis exuviation. The periodic ecdysis primarily orchestrated by ecdysone and juvenile hormone is a distinct characteristic in life cycle of silkworm [73]. Abundant expressions of miRNAs at MLS implicated that miRNAs may be fine-tuning this complicated and transient process. This result also coincides with the expression pattern of bmo-let-7 reported previously [74], which revealed that the expression level of bmo-let-7 is higher at the beginning of each molt than at other periods. According to our results, bmo-miR-31, its homolog dme-miR-31a is expressed in a pair-rule pattern of 14 stripes and in the foregut, anterior endoderm, and hindgut [42], and miR-31 was also found to have a high expression level in the small intestine of mammals [75], which contributes to the tumorigenesis and the acquisition of a more aggressive phenotype in human colorectal cancer [76]. Those findings provided clues that bmo-miR-31 at MLS may control epithelial metabolism during molting. Another MLS-biased miRNA, bmo-miR-278, was also found at MLS, and its homolog dme-miR-278 was proven to control energy homeostasis in *Drosophila*, since miR-278 mutants have elevated insulin production and elevated circulating sugar [77]. The relatively high expression level suggested that bmo-miR-278, in company with synchronized expressions of bmo-miR-77, bmo-miR-10 and bmo-miR-14, may play a similar role in regulating energy metabolism at molting stage, compromising for the conflict of energy-hungry process and fasting behavior by targeting insulin receptor-like protein precursor (BIR), bombyxin, and BmCF1.

Our cloning and real-time PCR results showed the expression of miRNAs is outstanding in stage of MLS, both in sorts and quantities. These discoveries shed lights on the fine-tuning mechanism of miRNAs in regulating different developmental stages of *B. mori*. Further investigations are needed to expand our comprehensive understanding of the modulating networks of miRNAs in the development of *B. mori* and even other insects.

## Materials and Methods

### Experimental materials from silkworms

Our starting materials were from HCl-treated diapaused eggs (they are actually developing embryos but here we used the common word egg instead) of Chinese silkworm strain *Dazao* provided by the Sericultural Research Institute, Chinese Academy of Agricultural Sciences, Zhenjiang, P. R. China. Eggs were incubated at 25±1°C under illumination, and larvae were reared on an artificial diet produced by the Sericultural Research Institute of Shandong, P. R. China. Eggs were incubated at 25°C for 30 days, and then transferred to 4°C and kept cold for 2 months to terminate diapause. All developmental stages of the silkworm were obtained in the following manner: (1) eggs laid within a 20-hour period for pre-diapause stage (PDS), (2) eggs laid within 40–48 hours for diapause stage (DS), (3) chilled eggs just before revival for diapause-broken stage (DBS), (4) eggs collected 72 hours after diapause-broken stage for blastokinesis stage (BKS), (5) eggs collected 120 hours after diapause-broken stage for trachea-appearing stage (TAS), (6) eggs collected 200 hours after diapause-broken stage for bluish stage (BS), (7) larvae hatched at the first day were collected for newly-hatched larva stage (NLS), (8) larvae collected on day 3 of the fourth-instar for fourth-instar larva stage (FLS), (9) larvae collected at fourth-molting for molting larva stage (MLS), (10) larvae collected on day 4 of the fifth-instar for late fifth-instar larva stage (LFLS), (11) larvae collected at spinning for spinning stage (SS), (12) larvae collected 2 day after cocooning for pre-pupa stage (PPS), (13) pupas collected 6 days after cocooning for pupa stage (PS), and (14) moths collected before mating for moth stage (MS).

### Computational prediction of microRNAs

We used *Srnaloop* to predict putative miRNAs from the silkworm genome. The genome assembly and some functional annotations were downloaded from SilkDB (http://silkworm.genomics.org.cn/) and Silkworm genome research program (http://sgp.dna.affrc.go.jp/index.html). The optimized parameters for *Srnaloop* program were described previously [60], except for the parameter “-l 90”, which was specific to identify hairpins shorter than 90 bases and was based on observations on known insect pre-miRNAs. We screened those hairpin sequences using overlapping filter developed by Li *et al* [60]. For removing other non-coding RNAs, we used Rfam database (http://www.sanger.ac.uk/Software/Rfam/) and assembled a database of rRNA, tRNA, snRNA, snoRNA sequences by querying GenBank (http://www.ncbi.nih.gov/Genbank/index.html), with the appropriate feature keys (*D. melanogaster*, *D. pseudoobscure*, *A. mellifera*, *B. mori* and *A. gambiae*) as what in the insect ncRNA database. We also used a data set of silkworm CDS sequences (http://silkworm.genomics.org.cn/) to screen out protein-coding sequences.

Two strategies were taken to predict conserved and novel miRNAs. The first strategy for obtaining homologs or orthologs of previously validated miRNAs, and candidate hairpins were searched against sequences of known metazoan miRNAs in miRBase (release 10.0) [78]. Based on the nomenclature of miRNAs, newly identified miRNAs with less than two bases variations against known miRNAs were classified into the same miRNAs family.

The second strategy for predicting novel miRNAs in the remaining hairpin sequences is to reduce the number of false predictions of pre-miRNAs based on *Srnaloop*. We applied several additional sequence/structure filters such as GC content, minimal free energy of the full-length hairpin (hairpin mfe), minimal free energy of the core hairpin structure (core mfe), and the ratio of core mfe to hairpin mfe (ch_ratio) to select authentic pre-miRNAs as described previously [60]. We also investigated features of known mature miRNAs and their precursors of *D. melanogaster*, *D. pseudoobscure*, *A. mellifera*, *B. mori* and *A. gambiae*. The candidate hairpins with sequence and structural features within the optimized reference range values of insect miRNAs were extracted for further analysis.

To assemble our own predicted miRNAs database, we searched putative pre-miRNAs against genomic sequences of *D. melanogaster*, *D. pseudoobscure*, *A. mellifera* and *A. gambiae* from UCSC (http://genome.ucsc.edu/) and silkworm EST sequences downloaded from NCBI dbEST (http://www.ncbi.nlm.nih.gov/dbEST/index.html) for sequence conservation and analyzed their expression levels. We extracted the conserved pre-miRNAs whose 5′ arm or 3′ arm have ≥20-nt continuous fragments identical to a subject sequence and the opposite arm have at least 15-nt fragments matching the same subject sequence.

### Cloning of small RNAs

The isolation and enrichment procedure for small RNA fraction were performed by using the *mir*Vana™ miRNA Isolation Kit (Ambion, Austin, TX), and isolated small RNAs were further separated with flashPAGE™ Fractionator (Ambion, Austin, TX) according to the manufacturer's manuals. Small RNAs was cloned with special adaptors. The 3′ adapter oligonucleotide (5′-pUUUctatccatggactgtx-3′, where uppercase, lowercase, p, and x stand for RNA, DNA, phosphate, and inverted deoxythymidine, respectively) with a 5′ monophosphate and a 3′ inverted deoxythymidine to prevent self-ligation was ligated first to the small RNA preparation with T4 RNA ligase (NEB) at 37°C for 1 h. The ligation product was then recovered after fractionation and ligated to the 5′ adapter (5′-tgggaattcctcactAAA-3′). The ligation product was fractionated again and reverse-transcribed by using a RT primer (5′-TACAGTCCATGGATAGAAA-3′), followed by PCR amplification with the reverse (RT primer) and forward (5′-CATGGGAATTCCTCACTAAA-3′) primers. The gel-purified PCR products were finally ligated to pGEM-T vector and transformed into ElectroMAX^TM^DH10B^TM^ competent cells (Invitrogen).

### Sequence analysis

We used PHRED, CROSS_MATCH, and BLASTN for automated base calling, vector removal, and sequence comparison, respectively [79], [80]. After trimming off low-quality sequences, we compared miRNA candidates ranging from 16nt to 40 nt in length to the silkworm genome sequence for putative origins, and those with perfect matches were searched against Rfam and our custom-curated insect ncRNA databases to remove sequences that are neither siRNAs nor miRNAs based on their sequence and structural features. We used the silkworm CDS database to identify and to remove sequences from degradations of mRNAs, and we also confirmed the candidate sequences by identifying query sequences among our predicted miRNAs and in miRBase 10.0. To get a prediction of the folding of these miRNAs, the candidate sequences along with 100-nt upstream and downstream flanking sequences were ran through Mfold [81].

### Stem-loop reverse-transcription PCR confirmation and Real-time PCR quantification

cDNA was synthesized from total RNA by using miRNA-specific stem-loop primers obtained from commercial service (Takara, Dalian). For quantification of known miRNAs, cDNA was synthesized according to the TaqMan MicroRNA Assay protocol (Applied Biosystems, Foster City, CA) except bmo-miR-1, bmo-miR-13, bmo-miR-14, bmo-miR-77, bmo-miR-263a, bmo-miR-275, and U6 snRNA, whose stem-loop primers also obtained from commercial service. All the stem-loop RT primers and gene specific primers were listed in Table S3. Reverse transcriptase reactions contained 20 ng of RNA samples, 50 nM stem-loop RT primer, 1× RT buffer, 0.25 mM each of dNTPs (Promega), 0.01M DTT (Invitrogen), 5 U/µl SuperScript™ II reverse transcriptase (Invitrogen) and 0.25 U/µl RNase Inhibitor (Promega). The 15-µl reactions were incubated in an Applied Biosystems 2720 Thermal Cycler in a 96-well plate for 30 min at 16°C, 30 min at 42°C, 5 min at 85°C and then held at 4°C.

The cDNAs were diluted 15 times to perform PCR for expression confirmation or real-time PCR for expression patterns analysis. PCR mixture containing 1 µl cDNA, 0.5 µM forward and reverse primers, 1× PCR buffer, 1.75 mM Mg^2+^, 0.25 mM each of dNTPs (Promega) and 1.25 U Taq polymerase (Fermentas). The 20 µl PCR reactions were performed using Applied Biosystems 2720 Thermal Cycler in 200 µl micro-tubes for 5 min at 95°C, followed by 35 cycles of 15 sec at 95°C, 30 sec at 58°C, and 30 sec at 72°C. PCR products were detected by electrophoresis with 3% agarose gel containing ethidium and photographed under UV light. Real-time PCR was performed using an ABI Prism® 7300 Sequence Detection system. The 20 µl PCR included 1.33 µl RT product, 1× TaqMan Universal PCR master mix and 1 µl primers and probe mix of the TaqMan MicroRNA Assay. The reactions were incubated in a 96-well optical plate at 95°C for 10 min, followed by 40 cycles of 95°C for 15 sec and 60°C for 10 min. All reactions were run in duplicate. The threshold cycle (Ct) is defined as the fractional cycle number at which the fluorescence passes the fixed threshold. For analyzing the expression patterns of bmo-miR-1, bmo-miR-13, bmo-miR-14, bmo-miR-77, bmo-miR-263a, bmo-miR-275 and U6 snRNA, real-time PCR was performed in an ABI Prism® 7300 Sequence Detection system with Quant SYBR Green PCR kit (TIANGEN, BJ) following the manufacturer's instructions. The 20 µl reactions including 1.33 µl RT product, 1× RealMasterMix (SYBR) and 0.5 µM forward and reverse primers were incubated in a 96-well optical plate at 95°C for 10 min, followed by 40 cycles of 95°C for 15 sec, 58°C for 30 sec and 70°C for 30 sec. Melting curves for each PCR were carefully monitored to avoid nonspecific amplifications. All reactions were run in duplicate.

### Normalization and data analysis

Since there has not been a standard control for expression normalization for miRNAs, we adopted a strategy using U6 snRNA as an internal control. Relative quantification (RQ) of each miRNA expression was calculated with 2^−ΔCt^ method, and the data were presented as log_10_ of RQ of target miRNAs. Results were visualized with GENESIS (Alexander Sturn, Institute for Genomics and Bioinformatics, Graz University of Technology).

### Statistical analysis of miRNA expression profiles

For each miRNA, one-tailed *t*-tests were applied to experimental replicates of each pair of stages to assess significance of differential expression. Stage that has the highest expression level was identified by times of rejection of null hypothesis that expression of tested stage is no higher than the other. And for certain miRNAs, group of stages of high expression level was defined by rejection of null hypothesis between lowest expression value in the group and highest one of the remaining. Nonparametric correlation coefficients between profiles of different stages were presented as Spearman's *ρ* to demonstrate relative similarities. *p*<0.05 was considered statistically significant. All related calculation was performed using the software MATLAB version 2007a.

### Target gene prediction for miRNAs

For miRNAs target gene prediction, we extracted 3′ UTRs of the silkworm (*Dazao* strain) UniGene which downloaded from NCBI UniGene database (http://www.ncbi.nlm.nih.gov/sites/entrezdbunigene) by using PITA program[82] that takes target accessibility on the interaction of miRNAs and their targets into account, was employed to predict miRNAs targets using default parameters. We selected and analyzed the target genes with ΔΔG≤−5 kcal/mol from the original predictions.

## Supporting Information

Table S1 Genomic locations of putative miRNAs predicted based on homology conservation comparison with known Metazoa miRNAs
(0.06 MB XLS)

Table S2 Conservation patterns, expression levels and genomic locations of putative miRNAs predicted based on Sequence & Structural features filters
(0.08 MB XLS)

Table S3 Sequences of stem-loop RT primers,forward primers and reverse primers
(0.09 MB DOC)
