# Supplementary material for: The Silkworm (Bombyx mori) microRNAs and Their Expressions in Multiple Developmental Stages
Source: PLoS One. 2008 Aug 20;3(8):e2997. doi: 10.1371/journal.pone.0002997 (PMC2500172; doi:10.1371/journal.pone.0002997)
Supplement: Table S3 — Sequences of stem-loop RT primers,forward primers and reverse primers (0.09 MB DOC) [file pone.0002997.s003.doc]

**Table S3 Sequences of stem-loop RT primers，forward primers and reverse primers**

| **miRNAs ID** | **Primer** | **Sequence** |
| --- | --- | --- |
| bmo-miR-1920 | RT | GTCGTATCCAGTGCAGGGTCCGAGGTATTCGCACTGGATACGACGAACTC |
|  | Forward | AATAGAGCGTGCGCGTAGC |
|  | Reverse | GTGCAGGGTCCGAGGT |
| bmo-miR-1921 | RT | GTCGTATCCAGTGCAGGGTCCGAGGTATTCGCACTGGATACGACACCTGG |
|  | Forward | ATGAGATTCAGCCTTGCG |
|  | Reverse | GTGCAGGGTCCGAGGT |
| bmo-miR-1922 | RT | GTCGTATCCAGTGCAGGGTCCGAGGTATTCGCACTGGATACGACTCTTAA |
|  | Forward | CGTGAGTTCGTCGTGGAT |
|  | Reverse | GTGCAGGGTCCGAGGT |
| bmo-miR-1923 | RT | GTCGTATCCAGTGCAGGGTCCGAGGTATTCGCACTGGATACGACGCCACG |
|  | Forward | GCGTAATCGCGTACCG |
|  | Reverse | GTGCAGGGTCCGAGGT |
| bmo-miR-2008a | RT | GTCGTATCCAGTGCAGGGTCCGAGGTATTCGCACTGGATACGACCGACTC |
|  | Forward | ATACGGCGAGAGGGACG |
|  | Reverse | GTGCAGGGTCCGAGGT |
| bmo-miR-2008b | RT | GTCGTATCCAGTGCAGGGTCCGAGGTATTCGCACTGGATACGACAACCCG |
|  | Forward | GCCAGGGACGCTCCTTAG |
|  | Reverse | GTGCAGGGTCCGAGGT |
| bmo-miR-2008c | RT | GTCGTATCCAGTGCAGGGTCCGAGGTATTCGCACTGGATACGACTCTAAG |
|  | Forward | AGCGAGAGGGACGCTC |
|  | Reverse | GTGCAGGGTCCGAGGT |
| bmo-miR-2009 | RT | GTCGTATCCAGTGCAGGGTCCGAGGTATTCGCACTGGATACGACTCACCA |
|  | Forward | GACGGGACCCGAAAGA |
|  | Reverse | GTGCAGGGTCCGAGGT |
| bmo-miR-2010 | RT | GTCGTATCCAGTGCAGGGTCCGAGGTATTCGCACTGGATACGACCAATTA |
|  | Forward | CGCCACTACGGAAAC |
|  | Reverse | GTGCAGGGTCCGAGGT |
| bmo-miR-1926 | RT | GTCGTATCCAGTGCAGGGTCCGAGGTATTCGCACTGGATACGACCCTTTT |
|  | Forward | GCGGCAGGAATTCTAAAGC |
|  | Reverse | GTGCAGGGTCCGAGGT |
| bmo-miR-2007 | RT | GTCGTATCCAGTGCAGGGTCCGAGGTATTCGCACTGGATACGACCGGCCA |
|  | Forward | GCGGTAAAAACGTGCG |
|  | Reverse | GTGCAGGGTCCGAGGT |
| bmo-miR-1924 | RT | GTCGTATCCAGTGCAGGGTCCGAGGTATTCGCACTGGATACGACCACTAC |
|  | Forward | TGATGTCCGCGGAGGT |
|  | Reverse | GTGCAGGGTCCGAGGT |
| bmo-miR-1925 | RT | GTCGTATCCAGTGCAGGGTCCGAGGTATTCGCACTGGATACGACGTCCAT |
|  | Forward | GCCGCTTTTCAACATGG |
|  | Reverse | GTGCAGGGTCCGAGGT |
| bmo-miR-1* | RT | GTCGTATCCAGTGCAGGGTCCGAGGTATTCGCACTGGATACGACTCCACA |
|  | Forward | GCCGCTGGAATGTAAAGAAG |
|  | Reverse | GTGCAGGGTCCGAGGT |
| bmo-miR-13* | RT | GTCGTATCCAGTGCAGGGTCCGAGGTATTCGCACTGGATACGACAACTCG |
|  | Forward | GCGGCTATCACAGCCATT |
|  | Reverse | GTGCAGGGTCCGAGGT |
| bmo-miR-14* | RT | GTCGTATCCAGTGCAGGGTCCGAGGTATTCGCACTGGATACGACTAGGAG |
|  | Forward | CGGGGCTCAGTCTTTTTCTC |
|  | Reverse | GTGCAGGGTCCGAGGT |
| bmo-miR-77* | RT | GTCGTATCCAGTGCAGGGTCCGAGGTATTCGCACTGGATACGACTGGACA |
|  | Forward | CGGCTCATCAGGCCATAG |
|  | Reverse | GTGCAGGGTCCGAGGT |
| bmo-miR-263a* | RT | GTCGTATCCAGTGCAGGGTCCGAGGTATTCGCACTGGATACGACCGTGAA |
|  | Forward | GCGAATGGCACTGGAAG |
|  | Reverse | GTGCAGGGTCCGAGGT |
| bmo-miR-275* | RT | GTCGTATCCAGTGCAGGGTCCGAGGTATTCGCACTGGATACGACCGCGAT |
|  | Forward | GCGCTCAGGTACCTGAAG |
|  | Reverse | GTGCAGGGTCCGAGGT |
| U6 | RT | GTCGTATCCAGTGCAGGGTCCGAGGTATTCGCACTGGATACGACACGATT |
|  | Forward | CCTGCGCAAGGATGAC |
|  | Reverse | GTGCAGGGTCCGAGGT |

* Those miRNAs were also performed real-time PCR for quantification analysis.
